# The effects of tempo and loudness variations during warm-up with music on perceived exertion, physical enjoyment and specific performances in male and female taekwondo athletes

**DOI:** 10.1371/journal.pone.0284720

**Published:** 2023-04-27

**Authors:** Ibrahim Ouergui, Arwa Jebabli, Hamdi Messaoudi, Slaheddine Delleli, Hamdi Chtourou, Anissa Bouassida, Ezdine Bouhlel, Emerson Franchini, Luca Paolo Ardigò

**Affiliations:** 1 High Institute of Sport and Physical Education of Kef, Kef, University of Jendouba, El Kef, Tunisia; 2 Research Unit: Sports Science, Health and Movement, UR22JS01, University of Jendouba, El Kef, Tunisia; 3 Institut Supérieur du Sport et de l’Education Physique de Sfax, Université de Sfax, Sfax, Tunisia; 4 Activité Physique, Sport et Santé, Observatoire National du Sport, Tunis, Tunisia; 5 Laboratoire de Physiologie de l’exercice et Physiopathologie, de L’intégré au Moléculaire "Biologie, Médecine, Santé", Faculty of Medicine Ibn El Jazzar, University of Sousse, Sousse, Tunisia; 6 Martial Arts and Combat Sports Research Group, School of Physical Education and Sport, University of São Paulo, São Paulo, Brazil; 7 Department of Teacher Education, NLA University College, Oslo, Norway; University of Split, CROATIA

## Abstract

The ergogenic effect of pre-selected warm-up music with the tempo and loudness variations on the performance of combat sports’ athletes as well as the difference between sexes is not well established. The present study aimed to assess the effects of listening to music with different tempos and loudness during warm-up on perceived exertion, physical enjoyment and physical performances in young taekwondo athletes. In a randomized study design, 20 taekwondo athletes (10 males, mean ± SD: age: 17.5 ± 0.7 years, taekwondo experience: ≥ 6 year) performed the taekwondo specific agility test (TSAT) and the 10s and multiple frequency speed of kick test (FSKT-10s and FSKT-mult) after warming-up with or without music. The music was played at high (140 beats·min^-1^) or very high (200 beats·min^-1^) tempo combined with low (60 dB) or high (80 dB) loudness, resulting in four experimental and control conditions. The ratings of perceived exertion (RPE) and physical activity enjoyment scale (PACES) were assessed after each condition. After normality, homogeneity and sphericity checks, two-way (or multivariate) analysis of variance and Bonferrroni (or Friedman’s and Wilcoxon’s test) post-hoc test were operated when necessary. For TSAT, 140 beats·min^-1^+80 dB induced better performance compared with 200 beats·min^-1^+80 dB, 200 beats·min^-1^+60 dB, control and the 140 beats·min^-1^+60 dB conditions. For FSKT-10s, 140 beats·min^-1^+80 dB condition induced higher performance compared with 200 beats·min^-1^+60 dB, 200 beats·min^-1^+80 dB, 140 beats·min^-1^+60 dB and the control conditions. For FSKT-mult, 140 beats·min^-1^+80 dB induced higher number of techniques compared with 200 beats·min^-1^+60 dB, 140 beats·min^-1^+60 dB, control and the 200 beats·min^-1^+80 dB conditions. Moreover, 140 beats·min^-1^+80 dB induced lower decrement index (DI) compared with the other conditions and lower DI in 140 beats·min^-1^+60 dB compared with 200 beats·min^-1^+80 dB and control conditions. Moreover, 140 beats·min^-1^+80 dB resulted in greater PACES scores compared with 200 beats·min^-1^+80 dB and control conditions. Better performance was found for males compared with females in TSAT, FSKT-10s and FSKT-mult (i.e., techniques’ number), as well as lower DI and higher RPE post-FSKT-10s. Pre-selected warm-up music played at 140 beats·min^-1^ and 80 dB is an efficient strategy to enhance physical activity enjoyment and specific performances in taekwondo.

## Introduction

Taekwondo is an Olympic combat sport characterized by intermittent high-intensity actions [1], which can lead to a high level of fatigue [2]. Therefore, to be successful in taekwondo athletes need to develop high physical abilities (i.e., agility, balance, coordination, speed, reaction time, and a high capacity to kick powerfully and repeatedly [1]) supported by contained rating of perceived exertion (RPE [3]). Thus, preparing athletes to cope with combat sports’ demands requires an exploration of various strategies that might improve both their mental and physical skills.

As an external stimulus, music has been investigated as a potential aid which regulates emotion and improves performance in a wide range of exercise modes and intensities [4–6]. The ergogenic (work-enhancing) potential of music is mainly based on its motivational properties, the reduction of fatigue perception (via attentional dissociation), and the regulation of affective arousal [4, 5], as well as the increase of positive mood, feelings of power, and exercise enjoyment [7, 8]. From a neuro-physiological perspective, music has been shown to delay neuromuscular fatigue and increase power production, implying improved muscle efficiency [9], neural activity [7, 10] and recovery state [11]. Music also promotes auditory-motor synchronization and rhythmic action [4, 11], resulting in higher levels of endurance, power, and strength [6, 12]. Overall, Karageorghis et al. [11] developed and validated with both aerobics teachers and exercise participants a 13-item and four-factor structure (association, musicality, cultural impact and rhythm response) accounting for 59.2% of the variance of the psychophysical responses to music listening. The original conceptual framework predicted that four factors would contribute to the motivational quality of music including association (how an individual interprets music), musicality (melodic and harmonic elements), cultural impact (socio-cultural background) and rhythm response (tempo and accentuation [13]). These variables were grouped into either internal (‘music factors’) or external (‘personal factors’ [13]). Through further research, these four factors were shown to be hierarchical, with rhythm response being most important [14].

Although there is a large body of evidence on the benefits of music listening in sport, these findings are not applicable in several sports (e.g., combat sports), since athletes from these modalities are not permitted to listen to music during competitions [15]. In this regard, it has been speculated that starting a training session or a competition in the best physical and emotional condition is paramount for success [11]. More specifically, it was shown that music is able to affect the central nervous system in terms of increased attention toward visual and auditory stimuli and more autonomous movements and decreased RPE [7, 10]. Pre-task music has been shown to bring back good memories [11] and improve athletes’ emotional state and self-efficacy [16]. An ergogenic effect of pre-task music has been evident on short and predominantly anaerobic tasks, including grip strength, Wingate test peak and mean power, and short-duration sports [e.g., 17]. However, some findings have been inconclusive in terms of ensuing ergogenic effects. While some studies showed that listening to pre-selected music during warm-up improved power output during a Wingate test, circuit-type resistance exercise and treadmill running performances [15, 18–21], another study did not report positive effects on power output during a supramaximal cycle exercise test [22].

One of the ways in which pre-task music has an effect on performance is through increased psychomotor activation [11]. The influence of music on athletes’ level of activation is closely dependent on its acoustical properties, such as tempo, rhythm, volume, lyrics and motor auditory synchronization [11, 23]. During high-intensity exercise, the loudness of musical stimulation becomes more important, as higher levels of activation are desired [7, 24]. Previous studies showed that the ergogenic effects of loud music on human behavior were attributed to its arousing properties [19, 24]. This is evident as athletes perceive loud music more enjoyable than less intense music [11]. Regarding music tempo, previous investigations showed that fast upbeat music would be suitable for fast power type activities [15, 23], while slow-tempo music might result in sedative effects [19]. In this regard, it has been suggested that any song with a tempo higher than 120 beats·min^-1^ can be considered stimulating and may enhance exercise performance [6, 23].

It has been suggested that the interactive effects between tempo and loud could determine athletes’ response to music [25]. Specifically, the interaction between high tempo and loudness was reported to improve running speed, grip strength, and preferred reaction time and reduced time to exhaustion [19, 25]. However, these responses were dependent on the athletes’ personality profile and the nature of the task in which they are engaged [21, 24]. In taekwondo, the use of music did not receive much interest. In this regard, Hammad et al. [3] showed that music tempo variations (i.e., slow: 80 beats·min^-1^, and fast: 200 beats·min^-1^) during the Wingate anaerobic test did not affect taekwondo athletes’ physiological responses, and their peak power output during the test. However, the lack of specificity of the testing procedure (i.e., the use of generic tests) and the timing of music listening (i.e., in-task) could limit the application of these findings to taekwondo performance.

Additionally, sex has been reported as an important factor which can modulate athletes’ responses toward music [26, 27]. Recent studies [27–29] showed that listening to music while exercising resulted in controversial differences between males and females. Results showed that there was a higher ergogenic effect of music in males than females [29], similar variation [28], or higher effect in females than males [27, 30]. In the aforementioned reports, sex effects were assessed using in-task self-selected music. To the current knowledge of the authors, there is only one previous investigation [20] which compared the ergogenic effects of warm-up pre-selected music between sexes and found no significant difference. However, this study was not specific to combat sports and consequently to taekwondo. Therefore, this study aimed at examining the effects of varying music tempo (140 beats·min^-1^ and 200 beats·min^-1^) and loudness (60 dB and 80 dB) of pre-selected warm-up music on perceived exertion, physical enjoyment and specific physical performances of male and female taekwondo athletes. As the interactive effect of a tempo>125 beats·min^-1^ and loudness >70 dB was reported to improve performance and arousal [25], our specific research question was whether listening to music with 140 beats·min^-1^ and 80 dB during warm-up could result in better performances.

## Materials and methods

### Participants

A priori power analysis was performed using the G*Power software (Version 3.1.9.4, University of Kiel, Kiel, Germany) using the F test family (ANOVA: repeated measures, within factors). Regarding taekwondo specific agility test performance and RPE assessment, the analysis revealed that a total sample size of 20 participants would be sufficient to find significant differences (effect size f = 0.25, α = 0.05) with an actual power of 80.23%. Following a convenience sampling, the participants were recruited from a local training club based on the following inclusions criteria: 1) have at least 6 years of experience; 2) do not suffer from hearing impairments; 3) be 15 to 18 years old; and 4) be in the early follicular phase, for female athletes. Twenty competitive taekwondo athletes (10 males and 10 females) participated in this study (Table 1). Athletes participating in the present study were all black belt. The study was conducted in the in-season period. The athletes were in good health, not engaged in any weight loss process and did not present injuries or hearing impairments during the experimentation. Moreover, due the fact that lifestyle background in terms of music listening experience may have a considerable effect on psychophysical reactions (i.e., improved mood, increased arousal control and decreased RPE) to music [12], a homogenous sample was gathered. Specifically, since music may occupy more attentional resources for people with stronger habitual music-listening behavior [31],habitual music-listeners were recruited through a verbal question on their music listening experience (did you listen to music daily?). Moreover, to control for the possible effects of the menstrual cycle, all female athletes were instructed to take part in the experiments when they were in their early follicular phase. Each female athlete was asked to indicate if she reached the menstruation phase and how long she had from the last day of menstruation (no more than 2 days to be included). Prior to proceeding, all participants were informed about the benefits and risks involved in the investigation and they and/or their parents signed a written consent. This study was carried out in accordance with the Declaration of Helsinki and the protocol was fully approved by the Southern Committee for Human Protection Tunisia (CPP SUD n° 0333/2021).

**Table 1:** Characteristics of the participants (values are mean ± standard deviation; n = 20).

Age (years)	Body mass (kg)	Height (cm)	Taekwondo experience (years)
17.5±0.7	59.2±10.0	168.1±9.5	7±1

### Design

**Fig 1:**
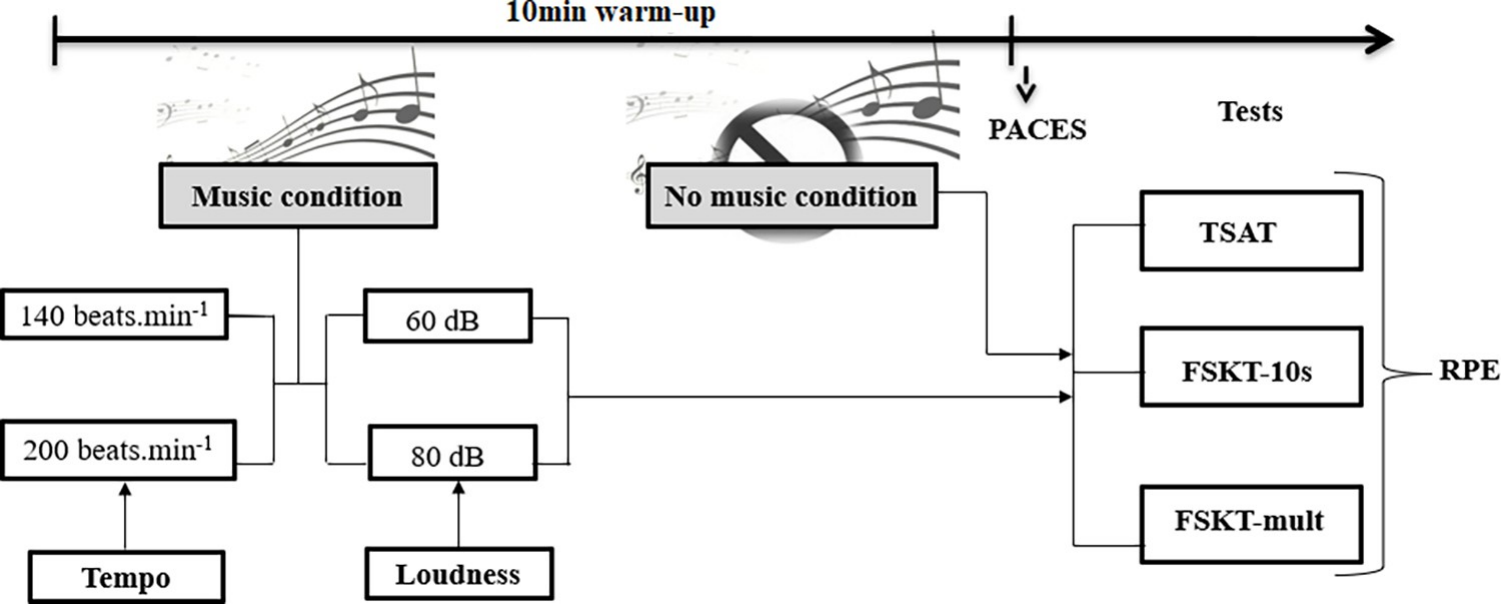
Schematic representation of the study design. PACES: Physical Activity Enjoyment Scale; RPE: Rating of perceived exertion; TSAT: taekwondo specific agility test; FSKT-10s: 10s frequency speed of kick test; FSKT-mult: multiple frequency speed of kick test.

One week before the beginning of the experimentation, athletes were well familiarized with the experimental procedures. The study followed a repeated measures/within-subjects study design in which all athletes completed all the experimental and control conditions. They were divided into five groups (to explain the random order) and familiarized with the testing protocols (the aim of the tests and the kick technique to use), the scales (timing and items) and the denouement of each condition. Therefore, participants performed five test sessions in a randomized and counterbalanced study design. All tests were performed at the same time of day in a gym with a moderate temperature (25–26°C). Same verbal encouragements were provided in each condition. In each test session, participants performed a standardized warm-up session, which is commonly used and well tolerated by both male and female athletes. The warm-up session involved running of 10 minutes at an average speed of 9 km·h^-1^with or without music. The pace was controlled a blinking light. Following this, in random order, participants completed the taekwondo specific agility test (TSAT [32]), the 10 s frequency speed of kick test 10s (FSKT-10s [33]) and its multiple version (FSKT-mult [34]). The whole testing session lasted around 10 min (including rest between tests). The choice of the 9 km·h^-1^ speed for the warm-up was based on previous studies [33–35] using the FSKT-10s and the FSKT-mult as testing procedures. Such warm-up speed could enable athletes to create an active synchronization (i.e., consciously synchronize movement rate with the rhythmical qualities of music) with the fast loud music, which could reduce the metabolic cost of the warm-up by promoting greater neuromuscular and kinetic efficiency [6, 36]. Athletes were instructed to rate their RPE [37] just after each test; while the physical activity enjoyment scale (PACES [38]) score was obtained after the warm-up session (Fig 1).

### Musical stimuli

The music, a tune previously chosen by one of the investigators (Jason Derulo, Get Ugly, 3:20), was played–looped–only during the 10-min warm-up, in respect to the requirement of the competitive conditions [15]. The track was selected based on the five recommendations of Karageorghis et al. [14]. Consequently, a pop fast music was selected to be used as stimulus in this investigation. To avoid any variation effect of the other intrinsic components of music (e.g., metric, melody and harmony [11]), the chosen track was played during all sessions. The decibel level of the music was administered under check using the application Decibel: dB sound level meter (developer Vlad Polyanskiy). Based on the fact that fast and loud music has been shown to induce the most positive effects on athletic performance and emotional states [7, 19, 20], especially in high-intensity exercise [39], high (140 beats·min^-1^) and very high (200 beats·min^-1^) tempos were used and combined with low (60 dB) and high (80 dB) volumes. The tempo of the selected music was changed using the Music Speed Changer software (Single Minded Productions, https://singlemindedproductions.com). Therefore, athletes were randomly assigned to the following conditions (with each condition on a different day): 1) warm-up music at 140 beats·min^-1^ and 80 dB, 2) warm-up music at 140 beats·min^-1^ and 60 dB, 3) warm-up music at 200 beats·min^-1^ and 80 dB, 4) warm-up music at 200 beats·min^-1^ and 60 dB and 5) warm-up without music (control condition). In the warm-up music conditions, music was played through personal headphones (without noise cancellation capability) connected to personal mobile phones and was turned off at the end of the warm-up. However, in the control condition, headphones were worn but no sound was played during the warm-up [39]. A sufficient period of passive recovery (~2-3min) between subsequent tests was allowed to athletes. All (condition) testing sessions were performed at the same time of day (17h00-19h00), with 48-h of rest interval in between [40].

### Testing procedures

#### Taekwondo specific agility test

In a guard position with both feet behind the start/finish line, the athlete moves as quickly as possible towards the center point, he/she turns towards partner 1 by performing a sideways movement and performed a roundhouse kick lead leg at his own choice (S1 Movie). After that, he/she turned to the other side and shifted to partner 2 (partners were other athletes not involved in that day session) and performed another roundhouse kick with the other lead leg, returned to the center moved forward to partner 3 in a guard position and performed a double roundhouse kick. Finally, the athlete runs backward to the start/finish line (for details, see Fig 1 from Chaabene et al. [32]). The completion time was measured using photocells (Brower Timing Systems, Salt Lake City, UT, USA). Three trials were performed by each athlete and the best one was used for analysis. Intraclass correlation coefficient (ICC) between test-retest trial was 0.85.

#### Ten seconds frequency speed of kick test

The test was performed as described by Da Silva Santos et al. [33]. The athlete was instructed to perform the maximum number of bandal-chagui kicking techniques (viz., a semi-circular kick performed with foot dorsum on the abdomen height of the opponent) alternating the left and right leg, executed on a punching bag, with the total number of kicks during 10 s (counted by two researchers (S2 Movie); in case of discrepancies, average count was taken into account) represented the performance index [33]. Three trials were performed by each athlete and the best one was used for analysis. Intraclass correlation coefficient for rest-retest trial was 0.76.

#### Multiple frequency speed of kick test

FSKT-mult was performed following the procedure during the FSKT-10s, where athletes performed 5 sets of the FSKT-10s with a 10 s of rest interval in between (S3 Movie). The number of kicks performed in each set and the total number of kicks in the 5 sets (counted by two researchers) were used to determine the performance index (DI; [34, Eq 1]):

DI(%)=[1‐((FSKT1+FSKT2+FSKT3+FSKT4+FSKT5)/(BestFSKTset×Numbersofsets))]×100 (1)

#### Rating of perceived exertion

Perceived exertion was assessed using the Borg CR 0–10 scale [37]. This scale ranging from “0” to “10”, with corresponding verbal expressions, that gradually increase with the intensity of perceived sensation (0 = Nothing at all; 0.5 = Extremely weak; 1 = Very weak; 2 = weak; 3–4 = Moderate; 4–5 = Strong; 5–6 = Severe; 7–9 = Very strong; and 10 = Extremely strong). Over sessions, athletes were shown a board with scores and descriptions and were asked to rate their RPE just after each test.

#### The physical activity enjoyment scale

This scale (PACES) was used to assess physical enjoyment [38]. Specifically, athletes were asked following question: “How do you feel at the moment about the physical activity you have been doing?”. The PACES inventory contains 8 items rated with a 7-point score ranging from 1 to 7. The PACES has 11 negative items and 7 positive items. Negative items are reverse-scored. For each participant, total responses are summed to give a score ranging from 18 to 126. Higher PACES inventory scores reflect greater levels of enjoyment. The internal consistency of this scale as measured by Cronbach’s coefficient alpha results 0.93 [38]. The PACES was already used to measure the physical enjoyment level featuring previous studies on combat sports like taekwondo [41] and judo [42, 43], which showed its ability to test this.

### Statistical analyses

The statistical analysis was performed using SPSS 20.0 statistical software (IBM corps., Armonk, NY, USA). Data were presented as mean and standard deviation, whereas only RPE values after FSKT-mult were presented as median and interquartile range. The Shapiro-Wilk test was used to check and confirm the normality of data sets, and the Levene test was used to verify the homogeneity of variances. Sphericity was tested using the Mauchly test. For the data normally distributed, a two-way analysis of variance (ANOVA; independent variables [condition × sex]) with repeated measurements was used to compare dependent variables (i.e., TSAT, FSKT-10s, PACES and RPE after TSAT and FSKT- 10 s) throughout the different experimental conditions, while the FSKT-mult outcomes (total number of techniques and decrement index) was compared using a multivariate analysis of variance (MANOVA). When the ANOVA indicated significant difference, Bonferroni was used as post-hoc test. Partial eta squared (*η*_*p*_^*2*^) effect size values were reported and classified as 0.01 = *small*, 0.09 = *medium*, 0.25 = *large* [44]. Moreover, standardized effect size analysis (Cohen’s *d*) was used to interpret the magnitude of differences between variables and considered as: *trivial* (*d* ≤ 0.20); *small* (0.20 < *d* ≤ 0.60); *moderate* (0.60 < *d* ≤ 1.20); *large* (1.20 < *d* ≤ 2.0); *very large* (2.0 < *d* ≤ 4.0); and *extremely large* (*d* > 4.0 [45]). In addition, the upper and lower 95% confidence intervals of the difference (95% CI) were calculated for the corresponding variation. However, for the RPE values after FSKT-mult where the data were not normally distributed, the comparison was conducted using the Friedman’s test and the Wilcoxon’s test was used as post-hoc. The *P*-value was set at 0.05.

## Results

### Taekwondo-specific agility test

There was a significant main effect of condition (*F*_4,6_ = 33; *P* < 0.001; *η*_*p*_^*2*^ = 0.96), with140 beats·min^-1^+80 dB condition resulting in lower TSAT time (i.e., faster TSAT accomplishment) compared to 200 beats·min^-1^+60 dB (95% CI [s] = -0.915 to -0.39; *d* = -2.12; *P* < 0.001), 200 beats·min^-1^+80 dB (95% CI = -0.844 to -0.255; *d* = -2.07; *P* = 0.001), 140 beats·min^-1^+60 dB (95% CI = -0.96 to -0.18; *d* = -1.43; *P* = 0.004) and the control (95% CI = -0.075 to -0.558; *d* = -2.84; *P* < 0.001) conditions (Table 2). Similarly, the results showed a significant main effect of sex (*F*_1,9_ = 112; *P* < 0.001; *η*_*p*_^*2*^ = 0.93), with lower TSAT time in males than in females (95% CI = -1.304 to -0.846; *d* = -2.93; *P* < 0.001). However, there was no significant interaction between condition and sex (*F*_4,6_ = 2.2; *P* = 0.192; *η*_*p*_^*2*^ = 0.59).

**Table 2:** Physical performances during the taekwondo-specific tests following the different conditions (values are mean ± SD; n = 20).

		140 beats·min^-1^+60 dB	140 beats·min^-1^+80 dB	200 beats·min^-1^+60 dB	200 beats·min^-1^+80 dB	Control	Overall
**TSAT (s)**	M	5.6±0.4	5.0±0.1	5.6±0.4	5.4±0.2	5.8±0.3	5.5±0.3*
	F	6.6±0.7	6.0±0.2	6.7±0.4	6.7±0.4	6.8±0.4	6.5±0.4
	Overall	6.1±0.5	5.5±0.2^†,¶,a,b^	6.1±0.4	6.0±0.3	6.3±0.4	6.0±0.4
**FSKT-10s (number of bandal-chagui kicks)**	M	26±1	28±2	26±3	26±2	25±3	26±2^£^
	F	24±1	26±1	24±1	24±1	22±1	24±1
	Overall	25±1	27±1^§,a,c,¥^	25±2	25±2	24±2	25±2
**FSKT-mult (number of kicks)**	M	113±5	123±4	112±10	115±7	110±7	115±7*
	F	108±5	115±4	105±4	108±5	103±7	108±5
	Overall	111±5	119±4^†,c,$,d^	109±7	112±6	106±7	111±6
**FSKT-mult (DI, %)**	M	9±2	5±2	10±3	12±3	12±2	10±2^#^
	F	10±2	8±2	11±2	12±1	13±1	11±2
	Overall	10±2^$,†^	6±2^†,¶,+,c^	11±3	12±2	13±2	10±2

^†^ main effect of condition: higher than control condition (*P* < 0.001)

^§^ main effect of condition: higher than control condition (*P* < 0.05)

^¥^ main effect of condition: higher than 200 beats·min^-1^+60 dB (*P* < 0.05)

^¶^ main effect of condition: higher than 200 beats·min^-1^+60 dB condition (*P* < 0.001)

^a^ main effect of condition: higher 200 beats·min^-1^+80 dB condition (*P* = 0.001)

^$^ main effect of condition: higher than 200 beats·min^-1^+80 dB condition (*P* < 0.05)

^+^ main effect of condition: higher than 200 beats·min^-1^+80 dB condition (*P* < 0.001)

^b^ main effect of condition: higher than 140 beats·min^-1^+60 dB (*P* < 0.05)

^c^ main effect of condition: higher than 140 beats·min^-1^+60 dB (*P* < 0.001)

^d^ main effect of condition: higher than 140 beats·min^-1^+60 dB (*P* = 0.001)

* main effect of sex: higher than females (*P* < 0.001)

^£^ main effect of sex: higher than females (*P* = 0.001)

^#^ main effect of sex: better than females (*P* < 0.05); TSAT: taekwondo-specific agility test; FSKT-10s: 10s frequency speed of kick test; FSKT mult: multiple frequency speed of kick test; DI: decrement index; M: male; F: female; a.u.: arbitrary unit; n = number of techniques

### Ten seconds frequency speed of kick test

There was a significant main effect of condition on the number of kicks (*F*_4,6_ = 17.6; *P* = 0.002; *η*_*p*_^*2*^ = 0.92), with 140 beats·min^-1^+80 dB condition resulting in higher performance compared to 200 beats·min^-1^+60 dB (95% CI [number of bandal-chagui kicks] = 0.121 to 4.079; *d* = 1.06; *P* = 0.035), 200 beats·min^-1^+80 dB (95% CI = 0.908 to 3.292; *d* = 1.30; *P* = 0.001), 140 beats·min^-1^+60 dB (95% CI = 0.938 to 2.262; *d* = 1.20; *P* < 0.001) and the control (95% CI = 1.204 to 5.096; *d* = 1.63; *P* = 0.002) conditions (Table 2). Similarly, a significant main effect of sex was found (*F*_1,9_ = 21.7; *P* = 0.001; *η*_*p*_^*2*^ = 0.71), with higher performance in males than in females (95% CI = 1.234 to 3.566; *d* = 1.25; *P* = 0.001). However, there was no significant interaction between condition and sex (*F*_4,6_ = 0.7; *P* = 0.609; *η*_*p*_^*2*^ = 0.33).

### Multiple frequency speed of kick test (FSKT-mult)

#### Total number of kicks

There was a significant main effect of condition on the total number of kicks (*F*_4,90_ = 11.8; *P* < 0.001; *η*_*p*_^*2*^ = 0.35), with 140 beats·min^-1^+80 dB condition resulting in higher number compared to 200 beats·min^-1^+60 dB (95% CI [number of kicks] = 4.744 to 15.956; *d* = 1.81; *P* < 0.001), 200 beats·min^-1^+80 dB (95% CI = 1.694 to 12.906; *d* = 1.39; *P* = 0.003), 140 beats·min^-1^+60 dB (95% CI = 2.594 to 13.806; *d* = 1.77; *P* = 0.001) and the control (95% CI = 6.894 to 18.106; *d* = 2.21; *P* < 0.001) conditions (Table 2). Similarly, there was a significant main effect of sex (*F*_1,90_ = 30.8; *P* < 0.001; *η*_*p*_^*2*^ = 0.26), with males presenting higher performance compared to females (95% CI = 4.392 to 9.288; *d* = 1.16; *P* < 0.001). However, there was no significant interaction effect between condition and sex (*F*_4,90_ = 0.2; *P* = 0.954; *η*_*p*_^*2*^ = 0.01).

#### Decrement index

There was a significant main effect of condition (*F*_4,90_ = 26.1; *P* < 0.001; *η*_*p*_^*2*^ = 0.54), with 140 beats·min^-1^+80 dB condition inducing lower DI compared with the 200 beats·min^-1^+60 dB (95% CI = -6.495 to -2.466; *d* = -2.03; *P* < 0.001), 200 beats·min^-1^+80 dB (95% CI [%] = -7.77 to -3.741; *d* = -3.02; *P* < 0.001), 140 beats·min^-1^+60 dB (95% CI = -5.278 to -1.249; *d* = -1.56; *P* < 0.001) and the control (95% CI = -8.408 to -4.378; *d* = -1.57; *P* < 0.001) conditions (Table 2). Moreover, 140 beats·min^-1^+60 dB condition resulted in lower DI compared to 200 beats·min^-1^+80 dB (95% CI = -4.506 to -0.477; *d* = -1.13; *P* = 0.006) and control (95% CI = -5.144 to -1.115; *d* = -1.43; *P* < 0.001) conditions. Similarly, a significant main effect of sex was reported (*F*_1,90_ = 8.2; *P* = 0.005; *η*_*p*_^*2*^ = 0.08), with lower DI in males than in females (95% CI = -2.147 to -0.388; *d* = -0.59; *P* = 0.005). However, no significant interaction effect between condition and sex was found (*F*_4,90_ = 0.8; *P* = 0.524; *η*_*p*_^*2*^ = 0.04).

#### Ratings of perceived exertion

For TSAT, there was no significant main effect of condition (*F*_4,6_ = 0.4; *P* = 0.053; *η*_*p*_^*2*^ = 0.75), sex (*F*_1,9_ = 0.0; *P* = 0.95; *η*_*p*_^*2*^ = 0.00) or interaction between condition and sex (*F*_4,6_ = 2.5; *P* = 0.156; *η*_*p*_^*2*^ = 0.62; Table 3). For FSKT-10s, there was no significant main effect of condition (*F*_4,6_ = 4.4; *P* = 0.808; *η*_*p*_^*2*^ = 0.21). However, there was a significant main effect of sex (*F*_1,9_ = 5.7; *P* = 0.041; *η*_*p*_^*2*^ = 0.39), with higher scores after the test in males than females (95% CI [arbitrary unit] = 0.036 to 1.334; *d* = 0.61; *P* = 0.041). However, there was no significant interaction between sex and condition (*F*_4,6_ = 1.2; *P* = 0.39; *η*_*p*_^*2*^ = 0.45). For FSKT-mult, there was a significant main effect of condition (χ^2^(4) = 11.396, *P* = 0.022), with the control condition elicited lower RPE values compared to 200 beats·min^-1^+80 dB and 140 beats·min^-1^+80 dB (*P* = 0.028 and 0.017, respectively).

**Table 3:** Rating of perceived exertion (RPE) and physical enjoyment variations across the different conditions (n = 20).

			140 beats·min^-1^+60 dB	140 beats·min^-1^+80 dB	200 beats·min^-1^+60 dB	200 beats·min^-1^+80 dB	Control	Overall
**RPE (a.u)**	Post-TSAT	M	4.0±0.8	3.6±0.7	3.9±1.3	4.6±1.1	3.8±1.0	4.0±1.0
		F	3.7±1.2	4.2±1.3	3.9±0.9	4.1±1.1	4.1±1.7	4.0±1.2
		Overall	3.8± 1.0	3.9±1.0	3.9±1.1	4.3±1.1	3.9±1.4	4.0±1.1
	Post-FSKT-10s	M	5.2±1.2	4.9±0.7	5.6±1.0	5.6±1.3	5.4±0.8	5.3±1.0
		F	4.3±1.4	5.0±1.3	4.4±0.7	4.6±1.4	5.0±1.2	4.7±1.2^#^
		Overall	4.7±1.3	4.9±1.0	5.0±0.8	5.1±1.3	5.2±1	5.0±1.1
	Post-FSKT-mult†	M	8.0 (7.2;8.7)	8.0 (8.0;8.7)	8.0 (8.0;9.0)	8.0 (8.0;9.0)	7.5 (7.0;8.0)	8.0(8.0;9.0)
		F	7.0 (6.2;8.0)	8.8 (7.0;8.0)	7.5 (6.0;8.0)	7.0 (6.0;8.0)	7.0 (6.0;8.0)	7.0(6.0;8.0)
		Overall	8.0 (7.0;8.2)	8.8 (7.0;8.0)	8.0 (7.0;9.0)	8.0 (7.0;9.0)	7.0 (7.0;8.0)	8.0(7.0;8.0)
**PACES (total score)**		M	68±8	77±5	72±10	67±7	65±5	70±7
		F	70±18	71±8	69±15	62±11	60±12	66±13
		Overall	69±13	74±6^$^^,^^§^	70±13	64±9	63±9	68±10

^†^ values for RPE post-FSKT-mult are reported as median and interquartiles (median (interquartile 1; interquartile 3])

^#^ main effect of sex: lower than males (*P* < 0.05)

^$^ main effect of condition: higher than 200 beats·min^-1^+80 dB condition (*P* < 0.05)

^§^ main effect of condition: higher than control condition (*P* < 0.05); RPE: Rating of Perceived Exertion; PACES: Physical Activity Enjoyment Scale; TSAT: taekwondo specific agility test; FSKT-10s: 10s frequency speed of kick test; FSKT-mult: multiple frequency speed of kick test; M: male; F: female; a.u.: arbitrary unit.

### The physical activity enjoyment scale

There was a significant main effect of condition (*F*_4,6_ = 7.1; *P* = 0.019; *η*_*p*_^*2*^ = 0.83), with 140 beats·min^-1^+80 dB condition resulting in higher scores compared to 200 beats·min^-1^+80 dB (95% CI = 2.482 to 16.318; *d* = 1.20; *P* = 0.007) and the control (95% CI [arbitrary unit] = 3.361 to 18.839; *d* = 1.43; *P* = 0.005) conditions (Table 3). However, there was no significant main effect of sex (*F*_1,9_ = 0.9; *P* = 0.371; *η*_*p*_^*2*^ = 0.09), or interaction between sex and condition (*F*_4,6_ = 0.8; *P* = 0.588; *η*_*p*_^*2*^ = 0.34).

Full raw data are provided in S1 Table.

## Discussion

This study aimed at investigating the effect of different music tempos (i.e., 140 beats·min^-1^ and 200 beats·min^-1^) and volumes (i.e., 60 dB and 80 dB) of pre-selected warm-up music on specific physical performances, perceived exertion and physical enjoyment in young taekwondo athletes. The present study showed that 140 beats·min^-1^+80 dB condition resulted in better performance in the FSKT-10s, FSKT-mult and TSAT compared to the 140 beats·min^-1^+60 dB, 200 beats·min^-1^+60 dB, 200 beats·min^-1^+80 dB and the control conditions. Moreover, significant differences were reported between males and females for the physical performances and the PACES scores.

Previous studies examining the effects of listening to music during warm-up sessions on anaerobic performance have shown that fast music, with a tempo of > 120 to 140 beats·min^-1^, resulted in greater anaerobic power in the subsequent Wingate test [15, 20, 46] and short high-intensity sprint exercises [39]. This improvement is reasonable since fast and arousing music is suggested to be most suitable when an athlete needs to perform high-intensity movements [20]. Eliakim et al. [20] showed that listening to arousing music during warm-up increased pre-exercise heart rate, as a metabolic primer, and following Wingate test it improved mechanical power [20]. Moreover, Edworthy and Waring’s [19] found that very fast (200 beats·min^-1^) and loud (80 dB) music was an effective strategy to increase running speed. Nevertheless, the recreationally active individuals recruited in the study of Fox et al. [21] did not show performance enhancement during the Wingate test after listening to pre-selected warm-up music at 138 beats·min^-1^. Additionally, Marques et al. [47] found no differences during a sprint interval training session in terms of perceived exertion, affective responses, and mechanical power output between high-tempo self-selected music, randomly selected music, and no-music conditions in physically active males. Although, the influence of music on performance was reported to decrease significantly with increased fitness level [20], Fox et al. [21] reported that training specificity and fitness level may influence the relationship between music during warm-up and subsequent performance. This could be somewhat true as studies using well trained subjects [20, 39, 46] showed significant impact of warm-up music, while those recruiting active subjects [21, 47] did not. Relevant evidence showed that music brought greater pleasure and performance gain to the high than to the low tolerant participants even if both groups reported similar levels of enjoyment [48]. However, since music serves as an external source of motivation, training status is worthy of further exploration [6]. Iinconsistent findings such as the above could be also related to the task’s nature or the music’s intrinsic components such as rhythm, volume, type, genre, and melody [6, 21, 24]. This study demonstrated that listening to music at a tempo of 140 beats·min^-1^and a volume of 80 dB during warm-up induced beneficial effects on the physical performances of taekwondo athletes. Interestingly, the findings of the present study raise about the lack of improvement when a tempo of 200 beats·min^-1^ was used. It is possible to suggest that using a very high tempo, athletes may perceive music as a stress factor and anxiogenic stimulus [49]. Mayfield et al. [49] showed that listening to fast-paced music did improve cognitive task performance, but it also increased the distraction level, which is a proxy for anxiety [49].

In taekwondo, the high-intensity nature of the combat pushes athletes to repeat powerful strikes at high speed to score and react quickly to avoid the opponent attacks [1], leading to high level of fatigue [2]. Therefore, it is important for athletes to develop high-level physical skills to cope with competition schedules and manage physical effort well enough [3]. In this consideration, exercise enjoyment has been believed as a vital predictor of exercise adherence [8]. In the present study, the athletes’ preference for music during warm-up was demonstrated by the PACES scores. This was particularly evident when music was played at a tempo of 140 beats·min^-1^and a volume of 80 dB, despite the fact that the music was not self-selected. Both Chtourou et al. [15] and Jarraya et al. [46] have shown that warm-up while listening to music with a tempo ranging from 120 to 140 beats·min^-1^increased mechanical power during a subsequent Wingate test [15, 46]. Additionally, Karageorghis et al. [25] have shown that listening to pre-task music with a similartempo (i.e., 126 beats·min^-1^) can enhance affective valence following the task. Our results confirm these previous findings. However, Marques et al. [47] reported that enjoyment did not vary when listening to high-tempo self-selected music, randomly selected music and control conditions in physical active males during sprint interval training session.

During the FSKT-10 s and TSAT, taekwondo athletes are required to perform at high intensity, with a significant demand for anaerobic metabolic energy, for a short bout duration. As a sensory stimulus, music serves to increase neural activity and affective states (i.e., arousal, motivation, mood state) [7, 10] and this may lead to heightened sympathetic responses which could alter muscular power output. Specifically, the application of fast and loud music results in a neural state where individual scans his/her environment in a more vigorous manner, thereby identifying pertinent targets more rapidly [7]. Additionally, fatigue resulting from repeated high-intensity actions has been shown to impair mechanical output [2]. Thus, performance improvement during the multiple version of FSKT may confirm the effectiveness of listening to music in extending exercise durations at high-intensities [23]. Furthermore, pre-exercise fast-paced music has been shown to increase plasma epinephrine, which may potentiate the sympathetic response to exercise [22]. This response helps individuals cope with the consequent increased demands in physical, metabolic, respiratory and cardiovascular efforts [22]. In a study by Ghaderi et al. [50], listening to motivational music during a single circuit resistance exercise resulted in decreased lactate and cortisol changes, which may serve as an underlying mechanism for reducing fatigue [50]. The fact that these effects persisted despite the absence of music during the exercise might be explained by the athlete’s auditory imagery [7]. Consequently, the combination of physiological and psychological activation induced by music stimulus increased arousal [6], which could explain the performances’ improvement in the present study.

Fast and loud music would stimulate the listener by activating the central nervous system, independently of how the music is afterwards perceived [11]. In a functional magnetic resonance imaging (MRI) study, Bishop et al. [7] had young athletes lie in an MRI scanner while listening to fast-tempo, high-intensity music just before performing a reaction time task. They found that brain structures involved in reactive performance, particularly those dealing with visual perception, allocation of attention, and motor control, showed neural pre-activation. These findings support the results of the present study. Using electroencephalography measurement, music was reported to decrease brain connection between frontal and central cortex’ lobes (i.e., the sensorimotor regions), a phenomenon linked to reduced exercise consciousness and suppression of fatigue-related symptoms [10]. Within this context of complex psycho-physiological interactions, listening to music can elicit pleasant memories, induces an increase in motivation and enjoyment [51] and motor coordination [52, 53]. In other words, music seems distracting the brain’s attention away from fatigued muscles [5, 10]. Although this phenomenon is not yet completely understood, it may partially explain the decrease in fatigue rate observed in the present study, as indicated by the decreased DI in the FSKT-mult following the 140 beats·min^-1^+80 dB condition.

Considering perceived exertion, RPE appears to have a key impact on promoting effort after listening to warm-up music [18]. In this context, it was suggested that whether an individual likes or dislikes the listened music has been shown to affect mood states and RPE [4]. However, in the present study, findings revealed that there was no significant difference in RPE after listening to pre-selected warm-up music. This was in line with previous studies [15, 19, 21, 46, 47], that showed unchanged RPE scores during 30s supra-maximal sprint, sprint interval training session and running speed tests, even with different music’s tempo and loudness. The lack of changes in RPE scores over conditions (i.e., due to warm-up with music) could be related to the exercise intensity, as music is relatively ineffective in reducing RPE during very high intensities exercises [5, 11]. This is most likely related to the fact that self-pacing allows individual to maintain a targeted RPE level during exercise [21]. Stork et al. [8] suggested that the effects of music during high-intensity exercise may be somewhat neutralized by significant interceptive cues of physical discomfort associated with the exercise. However, this study’ results are inconsistent with those reported by previous investigation which showed a perceived exertion decrease after listening to music [18]. This discrepancy in results could be attributed to differences in exercise intensity, duration, and the music selection process [18].

Regarding the sex’ effects, we found greater performance in males than females with no significant interaction effect with conditions. Given that our measures were recorded in 4 among 5 conditions where music was used, this result prevents us to assume that the observed variations are due to the music stimuli. It was reported that muscular power (“The ability of a muscle or muscle group to exert a maximum amount of force in the shortest period of time”), neuromuscular fitness (“… refers to physical fitness components such as flexibility, muscle strength, muscle power, jumping ability, and speed.”), muscular endurance (“the ability to voluntarily produce force or torque repeatedly against submaximal external resistances, or to sustain a required level of submaximal force in a specific posture for as long as possible”), speed and agility were greater in male than female taekwondo athletes [1, 54, 55]. In combat sports aggressiveness is considered as a vital determinant of performance [56], aggression control was associated to hormonal adaptation to exercise [56] and, in particular, cortisol was shown to be higher in male than female [57]. Increased mobilization of energy reserves and aggression are associated with this hormone [57], which might explain performance differences between males and females. In addition, for cortisol is a stress hormone [57], the greater RPE values recorded in males than females, particularly after FSKT-10s, might be due to–at least partially–its effects. Yet, further investigation regarding this is strongly recommended [57].

The variation in sex’ physical responses to music stimulus shown in the above studies did not receive consensus from previous investigations using a warm-up music [20, 30] or in-task music [27, 58]. These studies reported that females showed greater benefits from listening to self-selected music. These greater benefits were recorded in higher repeated sprint performance [30], lower fatigue index during repeated high-intensity sprint exercise [27], and longer running distances during the 12 min Cooper test [58]. Reasonably, females may exhibit higher emotional sensitivity to musical stimuli compared to males [59]. According to neuro-imaging research, females who listen to music have different prefrontal brain activation and a better capacity to shift attention away from unpleasant thoughts than males [60]. However, these findings appear to be relative, as sex had no effect on exercise enjoyment in our investigation. Whereas, the above studies reported greater ergogenic effects of preferred music in females, other showed similar effects during the Wingate anaerobic test even within task music [28] or warm-up music [20]. For preference and timing of music listening could modulate the level of activation [17, 61], the observed discrepancy could be related to these features. Specifically, females may be more receptive to performance gains than males when listening to their preferred music but not when listening to non-preferred pieces [61]. Accordingly, sex differences in exercise responses in the context of warm-up music remain unclear and will require further exploration to precisely discover the mechanisms responsible for divergence.

The duration of the test phase is a feature that might account for the disparity in obtained results of previous studies [24]. In this regard, it is generally accepted that the effect of warm-up music is observed on short-term performance (e.g., Wingate and handgrip strength [15, 20, 25]). However, even with short-term anaerobic tasks, some investigations [21, 22] did not found an effect. This fact could be related the music temporal aspects, as musical intensity effects are considered immediate and a habituation effect emerges after a certain period of time, which reduce or even completely remove the impact of the sound pressure level over time [24]. However, Edworthy and Waring [19] showed that very fast (200 beats·min^-1^) and loud (80 dB) music was effective to improve running speed during 10-min treadmill exercise. In the case of our investigation, performing the tasks from shortest to longest after a 10-minute exposure to music was an effective strategy for extending the effects of music over the three tests. This could be due to the short duration of TSAT and FSKT-10s, which motivated athletes to do well, especially when adequate rest was provided between tests. In terms of the music temporal aspects effect, the current study found that when an adequate tempo and loudness interact, the effect of warm-up music may be greater.

Finally, we acknowledge some limitations in the present study. The main one is the failure to assess the subjective perceived activation induced by the warm-up pre-selected music. Furthermore, despite some indications on the positive effect of high-tempo music on endurance exercise performance [5], 200 beats·min^-1^ may be too fast and further research should better focus on other frequencies between 140 beats·min^-1^ and 200 beats·min^-1^. It is true, as well, that the interactive effects of fast tempo and high loudness has been reported to improve affective state and consequent performance [19]. Specially, during high-intensity exercise, high levels of activation are desired which required increased music intensity [7, 24]. Moreover, it was reported that fast upbeat music would be suitable for fast power type activities [15, 23] based on the mechanism that it increased levels of arousal and induced bodily actions that facilitate warm-up objectives. Therefore, due to the high intensity characteristics of the experimental tasks, the choice of intense and fast music was to verify if increasing the loudness (80 dB) and the tempo (200 beats·min^-1^) of warm-up music could result in larger effect on the subsequent performances. The choice of post-warm-up timing was to identify in which condition athletes started the testing session in the best psychological state, as this might determine the subsequent performance [11]. However, to optimize the assessment of different pre-taekwondo warm-up strategies (which might improve taekwondo exercise in terms of physical activity enjoyment), the physical activity enjoyment scale should be administered after each taekwondo test. Moreover, we did not investigate other music intrinsic components such as type and melody which may be the subject of future studies. We did not take into account either whether listened music was known to athletes or preferred by them. In addition, while the tests used in this research were specific to taekwondo, they did not reflect the cumulative psychological stress induced by competition. Therefore, further investigations are needed to verify whether music could affect performance and psychological well-being during taekwondo competition. Lastly, we acknowledge that we should have used a camera to count with more accuracy the kicks in the kicking tests.

## Conclusions

Listening to music at 140 beats·min^-1^ and 80 dB during warm-up session improved the physical activity enjoyment scores and the physical performances during specific tests within taekwondo athletes, without requiring athlete to exert additional effort during the warm-up. Therefore, over both the training warm-up or pre-competition phase, listening to music at 140 beats·min^-1^ tempo and 80 dB loudness is useful to improve the performance in some taekwondo-specific tests, proxies for overall performance.

## Supporting information

S1 Table Study’s raw data. (XLSX)

S1 Movie (MP4)

S2 Movie (MP4)

S3 Movie (MP4)
